# Wounds

**Published:** 1878-09

**Authors:** 


Wounds.—The most simple application for sealing up wounds is the old-fashioned tincture of benzoin, and it is the most successful. By it nearly all fresh wounds heal rapidly, while they do not do so under watery and fatty dressings. Tincture of benzoin has a remarkable property of uniting tissues and combining with blood. It is antiseptic, and, assisted by cotton-wool pads of lint and firm bandaging, will arrest hemorrhage from all vessels less in size than the radial artery. Non-recent wounds which suppurate it is not desirable to heal by adhesion. The most important item in the treatment of these is ventilation with as pure air as possible. None but the most evil results follow the application of waterproof materials, such as oiled silk and gutta percha tissue over the dressings. Such wounds invariably stink and slough; the wound is made unduly hot, products of decomposition are retained, the surface has a grayish, grumous aspect, and loses substance daily. A simple piece of lint or muslin covered by cerate, or dipped in lotions of Condy's fluid (1 to 40), or tincture of myrrh and water (1 to 20), spirit and water or weak carbolic acid lotion (1 to 60), with just a layer of bandage to retain the dressing in its place, is all that is necessary, save a daily syringing and washing with warm Condy's fluid and water. (Mr. Philip Cowan p. 124).—*Braithwaite's Retrospect of Med. and Surg.*

